# Nutrition optimization in enhanced recovery after brain tumor surgery: scoping review of evidence, gaps, and a proposed perioperative nutrition score

**DOI:** 10.1093/noajnl/vdag056

**Published:** 2026-04-03

**Authors:** Camilla Regalia, Tirone Young, Zerubabbel K Asfaw, Alejandro Carrasquilla, Michael E Ivan, Shawn L Hervey-Jumper, Walavan Sivakumar, Randy S D’amico, Isabelle M Germano

**Affiliations:** Department of Neurosurgery, Icahn School of Medicine at Mount Sinai, New York, NY, USA; Department of Neurosurgery, Icahn School of Medicine at Mount Sinai, New York, NY, USA; Department of Neurosurgery, Houston Methodist Hospital, Houston, TX, USA; Neurosurgery, Texas Back Institute, Plano, TX, USA; Department of Neurosurgery, University of Miami, Miami, FL, USA; Department of Neurosurgery, UCSF, San Francisco, CA, USA; Department of Neurosurgery, Pacific Neuroscience Institute, Santa Monica, CA, USA; Department of Neurosurgery and Neuroscience, Saint John’s Cancer Institute, Santa Monica, CA, USA; Department of Neurosurgery, Zucker School of Medicine at Hofstra/Northwell, Hempstead, NY, USA; Department of Neurosurgery, Icahn School of Medicine at Mount Sinai, New York, NY, USA

**Keywords:** brain tumor surgery, carbohydrate load, ERABTS, ERAS, nutrition protocol

## Abstract

**Background:**

Nutrition protocols are integral to enhanced recovery after surgery (ERAS), as inadequate nutrition is associated with adverse outcomes. Yet, evidence regarding the impact of specific nutritional interventions, particularly in brain tumor surgery (ERABTS), remains limited. We performed the first comparison of nutritional strategies in general ERAS versus ERABTS, to clarify outcomes and develop a structured perioperative nutrition score.

**Methods:**

We conducted a scoping review of PubMed, Scopus, and Embase (2020-2024) for ERAS protocols incorporating nutrition. A systematic literature review identified ERAS protocols with a nutrition focus. Data were categorized into general surgery ERAS and ERABTS cohorts. Pre- and postoperative nutritional strategies were analyzed in relation to outcomes.

**Results:**

A total of 2,908 patients across 22 ERAS studies and 1,802 patients across 14 ERABTS studies met inclusion criteria. Preoperative carbohydrate loading was implemented in 86% of ERAS and 64% of ERABTS studies; however, no consistent improvement in length of stay (LOS) or complications was demonstrated. Preoperative nutritional screening was performed in fewer than 50% of studies and often lacked a structured criterion. Postoperative interventions were inconsistently reported and poorly defined. Across ERABTS studies, favorable but heterogeneous outcomes included improved ambulation (50%), reduced ICU utilization (40%), and lower pain and nausea (25%).

**Conclusions:**

Our work highlights that nutritional elements frequently included in ERAS and ERABTS protocols are inconsistently defined and insufficiently linked to outcomes. To address this gap, we propose a perioperative nutrition score (PONS) to improve risk identification and stratification, as well as support tailored nutritional interventions in brain tumor surgery.

Key Points

- Nutritional elements are commonly included in ERAS and ERABTS protocols but remain inconsistently defined.
- Carbohydrate loading is a common element of ERAS and ERABTS protocols, albeit an associated outcome benefit is insufficiently documented.
- Preoperative nutritional assessment was performed in fewer than half of studies and rarely used structured criteria to define optimal nutrition.
- We propose a perioperative nutrition score (PONS) to identify high-risk patients and guide tailored interventions in brain tumor surgery.

Importance of the Study

Inadequate nutrition is a well-established risk factor for poor surgical outcomes, yet nutritional strategies within enhanced recovery after surgery (ERAS) protocols remain highly variable. In neurosurgical oncology, where patients often present with metabolic and functional vulnerabilities, the role of perioperative nutrition is particularly relevant but poorly defined. This study is the first to examine nutritional components of ERAS protocols in general surgery and brain tumor surgery (ERABTS), highlighting current practices and critical knowledge gaps. By comparing evidence from general surgery and neurosurgery, the study clarifies which interventions are commonly used yet lack consistent outcome benefits. Importantly, the findings underscore the absence of standardized approaches to nutritional assessment during perioperative care. This work provides a foundation for developing structured, evidence-based nutritional scales that can identify at-risk patients, enable risk stratification, and guide tailored interventions aimed at improving recovery and outcomes in brain tumor surgery patients.

## Introduction

Enhanced recovery after surgery (ERAS) protocols have transformed perioperative care by emphasizing multimodal strategies that reduce surgical stress, accelerate recovery, and improve patient outcomes.^1–8 ^Among these strategies, nutritional management is a cornerstone,^9^^,^^10^ as inadequate nutrition has been consistently linked with increased complications, prolonged hospital length of stays (LOS), and impaired functional recovery.^11–16^

While the first evidence-based ERAS consensus for colorectal surgery was published in 2005, the implementation of ERAS in neurosurgery remains in its early stages.^17^ Our group recently identified 8 evidence-based ERAS elements associated with improved outcomes in patients undergoing elective brain tumor surgery—referred to as enhanced recovery after brain tumor surgery (ERABTS).^18^ Nutritional strategies were identified as one of the core components.^18^

Despite the well-established association between nutritional status and surgical outcomes, evidence supporting specific nutritional protocols in neurosurgery remains limited.^10^^,^^19^ The absence of standardized perioperative guidelines is striking, given the unique metabolic challenges faced by neurosurgical patients. Brain injury and craniotomy trigger neuroendocrine stress responses leading to hypermetabolism, hyperglycemia, and protein catabolism, often compounded by prolonged operative times.^10^^,^^20^^,^^21^ That said, while brain injury and craniotomy are associated with a systemic hypermetabolic stress response, neuroimaging and metabolic studies demonstrate that cerebral glucose metabolism may be regionally reduced or globally depressed following injury, highlighting a dissociation between systemic and cerebral metabolic responses.^22–24^ Moreover, many patients undergoing brain tumor surgery present with brain metastases, which are 5 to 10 times more common than primary tumors.^25^ Patients with brain tumors often endure inadequate nutrition due to systemic cancer and prior treatments.^26^^,^^27^ These factors place neurosurgical patients at a particularly high need for nutritional optimization in the perioperative period.

Previous reviews have described nutritional considerations in surgical oncology broadly, but none have systematically compared ERAS protocols in general surgery with those applied to brain tumor surgery, nor proposed neurosurgery-specific risk stratification strategies. The aim of this study is to provide evidence-based data on which components of ERAS and ERABTS nutritional protocols most influence postoperative outcomes, and to identify existing knowledge gaps. We aim to characterize the nutritional elements of ERAS protocols in both general surgery and ERABTS; evaluate associations between nutritional interventions and postoperative outcomes; identify gaps limiting standardization; and propose a practical perioperative nutrition score (PONS) to support risk identification and tailored interventions in brain tumor patients.

By aligning neurosurgical perioperative care with broader ERAS principles while addressing the unique vulnerabilities of brain tumor patients, we aim to lay the groundwork for structured, evidence-based nutritional strategies that improve recovery and outcomes.

## Methods

### Scoping Review and Search Strategy

This review followed the Scoping review guidelines.^28^ A scoping literature search of PubMed, Scopus, and Embase was conducted for the period January 1, 2020, to December 31, 2024. Inclusion criteria comprised: 1) ERAS studies in adult patients that included preoperative carbohydrate loading or a nutrition-related protocol, and 2) studies written in English. Exclusion criteria included: 1) studies including exclusively diabetic patients, given the confounding effect of glucose control on nutrition protocols, 2) studies limited to gastrointestinal surgery, 3) literature reviews, and 4) case reports.

Search terms included: “Preoperative,” “Enhanced Recovery After Surgery,” “Nutrition,” “Adult,” “Carbohydrate Loading,” and “Surgery.” References were managed in Covidence (Veritas Health Innovation, Melbourne, Australia). Titles and abstracts were screened independently by 2 reviewers. Disagreements were resolved by the senior author. For ERABTS-specific protocols, previously published work^18^ served as the primary source.

This review was not registered in PROSPERO^29^ or another registry because it was designed as a descriptive synthesis of reported nutritional protocols rather than an interventional meta-analysis requiring prospective registration.

### Data Extraction and Data Analysis

Variables extracted from each study included: study year, country, surgical procedure, total patient number, ERAS/ERABTS cohort size, and nutrition protocol elements (eg cessation counseling, preoperative nutritional screening, carbohydrate loading, pre- and postoperative fasting guidelines, postoperative nutrition, and reported outcomes). Extraction was performed by one reviewer, and verified by a second for accuracy, with discrepancies resolved by consensus. No study investigators were contacted for additional data. Protocol elements were summarized as proportions of studies reporting each intervention: pre-operative carbohydrate loading, pre-operative solid food fasting, pre-operative liquid fasting, pre-operative nutritional assessment or counseling, post-operative nutritional protocols, smoking cessation counseling, alcohol cessation counseling, and mandatory smoking/alcohol cessation.

Microsoft Excel (Microsoft Corporation, Redmond, Washington) was used to compile key information on the nutritional protocols of each study as well as patient outcomes associated with such protocols.

### Statistical Analysis

Categorical data were summarized as counts and percentages; No continuous outcomes were pooled due to heterogeneity in reporting. Categorical variables were analyzed by Fisher’s exact test, 2-sided. A *P*-value < .05 was considered significant. All analyses were conducted using Python (SciPy v1.13.1, Statsmodels v0.14.2); descriptive tables and graphics were produced in Microsoft Excel 365.

### Quality Assessment

Because the primary aim was to map existing nutritional protocols rather than estimate treatment effect, a formal risk-of-bias tool (eg ROBINS-I, Newcastle–Ottawa) was not applied. This approach is consistent with PRISMA-ScR and related guidelines, which do not require risk-of-bias appraisal when the primary objective is to map existing evidence rather than to estimate treatment effects.^30^

## Results

### ERAS in General Surgery: Nutrition Protocols and Clinical Outcomes

**Figure 1.**
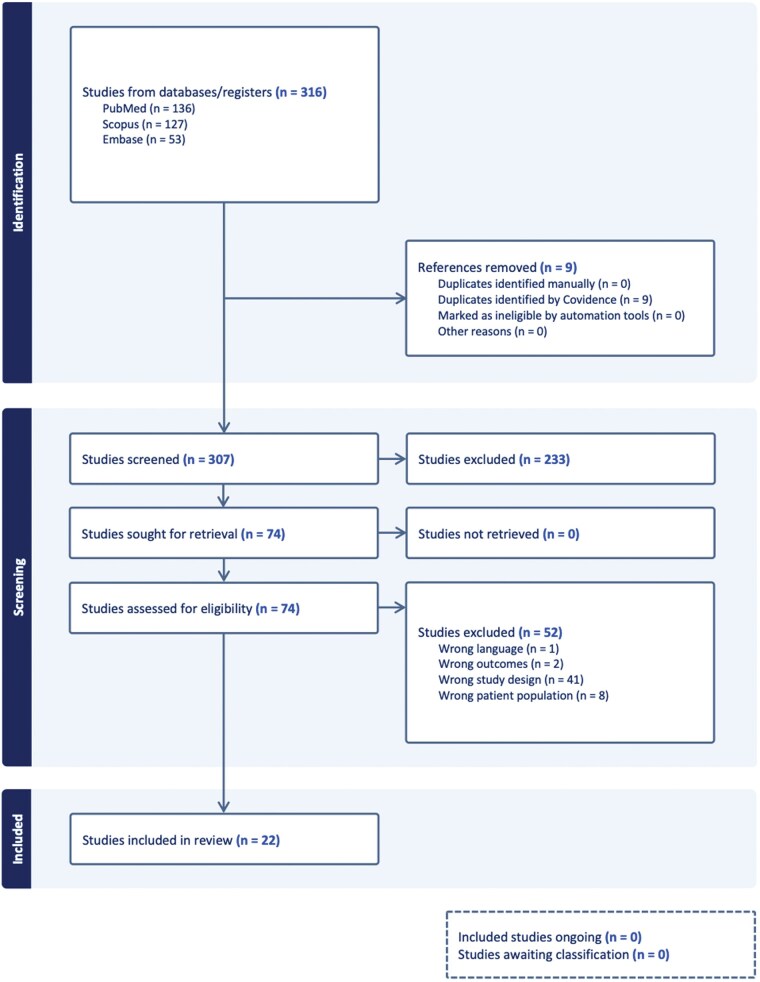
PRISMA flow diagram of the study selection process (generated via Covidence, www.covidence.org).

Of the 307 non-duplicated abstracts, 74 were accepted for full-text review, and 22 studies qualified for inclusion in this review (Figure 1). A total of 1,647 patients were included in ERAS protocols, with an additional 1,261 as controls, yielding a total of 2,908 patients across the 22 studies,^3^^,^^5–8^^,^^31–47^ undergoing a variety of surgical procedures, including but not limited to fracture fixations, elective cesarean sections, arthroplasty, and laparotomy (Tables 1 and 2). About a quarter of studies (23%) originated from the US (n = 5 studies, 749 ERAS patients).^3^^,^^32^^,^^34^^,^^36^^,^^40^

**Table 1.** Summary of key elements of ERAS nutritional protocols

Nutritional protocol key items	n = 22 (%)^a^
Pre-operative	
Carbohydrate loading^a^	19 (86%)^3^ ^,^ ^6–8^ ^,^ ^31–41^ ^,^ ^43–45^ ^,^ ^47^
Solid food fasting^a^	11 (50%)^8^ ^,^ ^31–34^ ^,^ ^37^ ^,^ ^39–41^ ^,^ ^46^ ^,^ ^57^
Liquid fasting^a^	10 (46%)^31–34^ ^,^ ^37^ ^,^ ^39–41^ ^,^ ^46^ ^,^ ^57^
Nutritional assessment or counseling^a^	18 (82%)^3^ ^,^ ^5^ ^,^ ^7^ ^,^ ^8^ ^,^ ^32–41^ ^,^ ^44–47^
Smoking cessation counseling	0 (0%)
Alcohol cessation counseling	0 (0%)
Mandatory smoking/alcohol cessation	2 (9%)^39^ ^,^ ^46^
Serum albumin measured	1 (5%)^7^
Post-operative	
Nutritional protocol^b^	7 (32%)^5^ ^,^ ^6^ ^,^ ^31^ ^,^ ^32^ ^,^ ^34^ ^,^ ^38^ ^,^ ^42^

a Described explicitly; Not including studies that may have implemented but did not write definitively in the protocol.

b Including specific indications when liquids and solids were to be consumed as well as polymeric drinks (n = 6)^5^^,^^6^^,^^31^^,^^32^^,^^38^^,^^42^ and serum albumin recordings (n = 1)^6^.

**Table 2.** ERAS nutritional protocols in general surgery

Author	Year	Country	Surgical procedure	ERAS patient N	ETOH/smoking cessation	Preoperative nutritional protocol	Preoperative fast (items/hrs pre-op)	Preoperative carbohydrate loading	Postoperative nutritional protocol
Akbuğa et al.^31^	2021	Turkey	Arthroscopic surgery	31 (51%)	Not specified	Not specified	Solid food/6; Clear fluids/2	400 mL sour cherry juice (200 kcal); 2 h	Pulpless, grain-free clear liquid; 2h
Alimena et al.^32^	2020	U.S.A	Laparotomy	302 (73%)	Not specified	Hyperglycemia screening; BMI noted	Solid food/midnight; Clear fluids/2	2 x 340 g maltodextrin; 2-3 h	Regular diet; day 0
Bai et al.^33^	2023	China	Laparoscopic hysterectomy	45 (50%)	Not specified	Not specified; BMI noted	Solid food/6; Water/6	Oral sugary electrolyte drinks (<400 mL); 2 h	Not specified
Chaudhary et al.^7^	2022	Nepal	Femur fracture fixation	33 (50%)	Not specified	Mini Nutritional Assessment Scale; Serum Albumin Recorded pre-op & post-op; Weight loss post-op & BMI noted	Not specified	100 g Glucose-D; Night before	Not specified
Chen et al.^5^	2022	China	Laparoscopic radical nephrectomy	40 (45%)	Not specified	Family meeting; BMI noted	Solid food/6; Water/2	N/A	6 h solid food fast
Cochran et al.^3^	2023	U.S.A	Hepatopancreatobiliary, radical cystectomy, head and neck procedures	297 (50%)	Not specified	Immune-modulating ONS; BMI noted	Not specified	296 mL Ensure^®^	Not specified
Elsabbagh et al.^34^	2022	U.S.A	Kidney transplant	20 (50%)	Not specified	Not specified; BMI noted; Serum Albumin Recorded pre-op	Solid food/6-8; Clear fluids/2	High carbohydrate clear drink was given; 2 h	Advance as tolerated.
Gumuskaya et al.^35^	2022	Australia	Laparoscopic cholecystectomy and thyroidectomy	68 (48%)	Not specified	Not specified; BMI noted	Not specified	60g of honey in 100 ml of water; 2 h	Not specified
Haselton et al.^36^	2025	U.S.A	Total Hip Arthroplasty	75 (50%)	Not Specified	Exclusion if BMI > 45 kg/m^2	Not Specified	8 oz. Ensure (maltodextrin); night before and 8 oz. Ensure (maltodextrin); 2 hr	Not Specified
He et al.^37^	2021	China	Elective cesarean	30 (33%)	Not specified; Implied	Not specified; BMI noted	Solid food/night before; Clear fluids/10 PM	400 ml oral solution (852 kJ caloric); 2 h	Not specified
Hendri et al.^38^	2021	Indonesia	Radical cystectomy	36 (59%)	MC (not specified)	Counseling (undescribed)	Fasting/6	Water with glucose 400 mg; 4 h	Fluid diet; 12h
Kotfis et al.^39^	2023	Poland	Elective cesarean	75 (51%)	Not specified; Implied	Not specified; BMI noted	Solid food/6; Clear fluids/2	200 mL 12.5% dextrose in water; 2 h	Not specified
Kuiper et al.^40^	2022	U.S.A	Laparoscopic Living Donor Nephrectomy	55 (37%)	Not Specified	Weight and BMI recorded	Solid Food/midnight Clear fluids/midnight	237 mL Ensure with 160 mL water (52 g); 2-4 h	Not Specified
Loodin et al.^41^	2021	Sweden	Hip fracture fixation	59 (54%)	Not specified	Screening for malnutrition risk; 6 mo. weight loss, problems eating, low BMI^a^	Solid food/6; Clear fluids/2	200-400 ml (47.5 g carbs/50 g and 190 Kcal)	Not specified
Nechay et al.^42^	2020	Russia	Laparoscopic Appendectomy	50 (48%)	Not Specified	Not Specified	Not Specified	Not Specified	Liquid intake/2 hr and Regular diet/6 hr
Minz et al.^43^	2024	India	Hip fracture fixation	30 (50%)	Not specified	Not specified	Fasting/6	200 mL of Aptonia powder (24g); 2 h and 400 mL coconut water, 50 g sugar; morning	Not specified
Van Loi et al.^8^	2025	Vietnam	Laparoscopic Gynecological Surgery	35 (50%)	Not Specified	Exclusion if BMI > 35 kg/m^2	Solid Food/10 pm night before	600 mL of 15% Maltodextrin; midnight and 300 mL of 15% Maltodextrin; 2 h	Not Specified
Walach et al.^45^	2024	Germany	Partial Nephrectomy	147 (48%)	Not Specified	Intravenous balanced fluid allowed overnight (1 ml/kg/h) before surgery	Not Specified	200 mL isotonic, 21% carbohydrate containing clear fluid; night before and 200 mL; 1 h and 3 drinks on first day post-op	Not Specified
Van Egmond et al.^44^	2023	Netherlands	Total knee arthroplasty	72 (51%)	Not specified	Not specified; BMI noted	Not specified	2 bottles of 200ml Nutrica Preop; 2-3 h	Not specified
Yi et al.^6^	2020	Malaysia	Gynecological surgery	62 (52%)	Not specified	Not specified	Fasting/midnight	474 mL (500 kcal, 100 g carbs, 18 g whey); 12 h and 237 mL (250 kcal, 50 g carbs, 9 g whey); 3 h	Omit solid food; 6 h and Clear formula; 4 h and Weight loss post-op noted; Serum Albumin Recorded post-op
Yilmaz et al.^46^	2020	Turkey	Minor gynecological procedures	51 (49%)	MC (4 weeks)	Not specified; BMI noted	Solid food/6; Clear fluids/2	N/A	Not specified
Zhang et al.^47^	2020	China	Ambulatory surgery	34 (53%)	Not specified	Physical exam and lab tests; BMI noted; Excluded BMI < 18.0 kg/cubic m	Fasting/8	200 ml of Outfast (112 kcal)	Not specified
			Total N:	1647					

Abbreviations: AEB, explained benefits of alcohol cessation; MC, mandatory cessation; SEB, explained benefits of smoking cessation.

a Peri-operative Nutrition Screen with the following criteria: BMI < 18.5 kg/cubic m, unintentional weight loss > 10% in 6 months, serum albumin <3.0 g/dL, poor oral intake for >= 7 days.

Preoperative assessment: nutritional screening was absent or vaguely described in half of the studies (11/22, 50%). Sixteen studies (73%) reported BMI, and only 2 (9%) recorded serum albumin. Counseling for smoking or alcohol cessation was rare (2/22, 9%).

Carbohydrate loading: 19 studies (86%) incorporated pre-operative carbohydrate loading protocols. The reported ERAS carbohydrate loading clinical outcomes are summarized in Table 3. Pre-operatively, reported benefits included reduced thirst (4/19, 21%) and reduced hunger (3/19, 16%). Post-operatively, reported benefits included reduced time to bowel movement (3/19, 16%), earlier return to diet (3/19, 16%), reduced pain (3/19, 16%), increased ambulation (3/19, 16%), and reduced nausea/vomiting (2/19, 11%). Two studies reported negative effects, in particular, increased pre-operative blood glucose^32^ and postoperative insulin resistance.^39^ Importantly, 3 studies that did not include carbohydrate loading also reported overall decreased LOS, suggesting benefit may derive from multimodal ERAS rather than carbohydrate loading alone.^5^^,^^42^^,^^46^

**Table 3.** Reported outcomes of nutritional and carbohydrates loading protocols in general surgery ERAS

Patient outcome	ERAS nutritional protocols n = 3 (%)	**Carb loading protocols n = 19 (%)** ^a^
Pre-operative		
Reduced thirst	0 (0%)	4 (21%)^8^ ^,^ ^39^ ^,^ ^43^ ^,^ ^47^
Reduced hunger	0 (0%)	3 (16%)^8^ ^,^ ^39^ ^,^ ^47^
Reduced anxiety	0 (0%)	1 (5%)^43^
Post-operative		
Reduced time to bowel movement	1 (33%)^46^	3 (16%)^33^ ^,^ ^34^ ^,^ ^38^
Reduced time to regular diet	1 (33%)^46^	3 (16%)^34^ ^,^ ^38^ ^,^ ^40^
Reduced pain	1 (33%)^42^	3 (16%)^7^ ^,^ ^34^ ^,^ ^36^
Increased ambulation	1 (33%)^46^	3 (16%)^7^ ^,^ ^34^ ^,^ ^38^
Reduced complications	1 (33%)^5^	1 (5%)^41^
Reduced nausea/vomiting	0 (0%)	2 (11%)^35^ ^,^ ^39^
Reduced glucose	0 (0%)	1 (5%)^31^
Reduced thirst	0 (0%)	1 (5%)^31^
Reduced insulin resistance	0 (0%)	1 (5%)^37^
Increased wound healing	0 (0%)	1 (5%)^33^
Increased blood lactate	0 (0%)	1 (5%)^39^
Increased mean modified barthel index	0 (0%)	1 (5%)^7^
Reduced time to drain removal	0 (0%)	1 (5%)^38^
Reduced orthostatic hypotension	0 (0%)	1 (5%)^44^
Reduced weight loss	0 (0%)	1 (5%)^6^
Increased muscle mass	0 (0%)	1 (5%)^6^
Overall		
Reduced length of stay	3 (100%)^5^ ^,^ ^42^ ^,^ ^46^	7 (37%)^3^ ^,^ ^6^ ^,^ ^7^ ^,^ ^34^ ^,^ ^36^ ^,^ ^38^ ^,^ ^40^
Reduced cost of stay	1 (33%)^5^	1 (5%)^3^
Prevention of decreased albumin	0 (0%)	1 (5%)^7^
Reduced readmission rate (<1 mo.)	0 (0%)	1 (5%)^6^

a Studies that included carbohydrate loading in their ERAS Nutritional Protocol.

Nine studies^3^^,^^48–55^ included patients with diabetes mellitus (DM) in their protocols. Of these, 2 reported data without differentiating between patients with and without DM,^3^^,^^50^ and 4^48^^,^^49^^,^^51^^,^^52^ did not assess outcomes against a control group or focused on the feasibility of an ERAS protocol rather than a comparative analysis of its implementation on patient outcomes. The key elements and nutritional protocols of the 3 remaining papers^53–55^ are summarized in Tables 4 and 5. Positive outcomes of carbohydrate loading protocols in DM patients included pre-operative reduced anxiety,^53^ post-operative reduced pain and increased ambulation,^54^ reduced post-operative glucose,^55^ and increased mean modified Barthel index.^53^

**Table 4.** Summary of key elements of ERAS nutritional protocols for diabetic patients

Nutritional protocol key items	**n = 3 (%)** ^a^
Pre-operative	
Carbohydrate loading^a^	3 (100%)^53–55^
Solid food fasting^a^	2 (66%)^54^ ^,^ ^55^
Liquid fasting^a^	2 (66%)^54^ ^,^ ^55^
Nutritional assessment or counseling^a^	3 (100%)^53–55^
Smoking cessation counseling	1 (25%)^53^
Alcohol cessation counseling	1 (25%)^53^
Mandatory smoking/alcohol cessation	0 (0%)
Serum albumin measured	0 (0%)
Post-operative	
Nutritional protocol^b^	2 (66%)^53^ ^,^ ^54^

a Described explicitly; Not including studies that may have implemented but did not write definitively in the protocol.

b Including specific indications when liquids and solids were to be consumed.

**Table 5.** ERAS nutritional protocols in general surgery for diabetic patients

Author	Year	Country	Surgical procedure	ERAS patient N	ETOH/smoking cessation	Preoperative nutritional protocol	Preoperative fast (items/hrs pre-op)	Preoperative carbohydrate loading	Postoperative nutritional protocol
Varughese et al.^55^	2024	India	Non-specific (“low to intermediate risk surgery”)	25 (50%)	Not Specified	Fasting plasma glucose and insulin noted at 5 AM on surgery day; BMI noted	Fasting/10 PM night before	47.5 g carbohydrate (190 kcal/kilojoules); 3 h	Not Specified
Qin et al.^53^	2024	China	Diabetic Foot Ulcer Surgery	55 (49%)	AEB, SEB	Nutritional support (non-specified) and correction of hypoproteinemia; Nutritional Risk Screening & Patient-Generated Subjective Global Assessment	No Fasting	Dynamic, based on patient’s individual differences; before surgery (non-specific)	Regular diet; Immediately
Elgamal et al.^54^	2023	Egypt	Elective Lumbar Decompression Surgery	36 (50%)	Not Specified	Oral hypoglycemic drugs discontinued after the last meal; BMI noted	Solid food/6; Clear fluids/4	400 ml carbohydrate drink (12.5 g/100 ml); 4 h	Oral intake; Immediately with insulin infusion until oral intake was established

### ERABTS: Nutrition Protocols and Clinical Outcomes

A total of 1,014 patients were included in ERABTS protocols, with an additional 788 controls, yielding 1,802 patients across the 14 studies.^56–69^ Of the 14 included studies, 21% (n = 3) involved patient cohorts in the U.S.^59^^,^^62^^,^^65^

Preoperative Assessment: Summary of key elements of ERABTS nutritional protocols and details are provided in Tables 6 and 7. Most protocols (12/14, 86%) included nutritional screening or counseling, though criteria were inconsistently defined. Six studies used vague descriptors such as “optimization” or “familial involvement.” Seven studies (50%) reported BMI, while only 2 (14%) measured serum albumin. Mandatory alcohol and smoking cessation were reported by 4 studies (29%), of the studies and 21% noted that the benefits of cessation were discussed with patients rather than mandating it.

**Table 6.** Summary of key elements of ERABTS nutritional protocols

Nutritional protocol key items	n = 14 (%)^a^
Pre-operative	
Carbohydrate loading^a^	9 (64%)^57^ ^,^ ^58^ ^,^ ^61^ ^,^ ^63^ ^,^ ^66–69^
Solid food fasting^a^	2 (14%)^61^ ^,^ ^66^
Liquid fasting^a^	2 (14%)^57^ ^,^ ^66^
Nutritional assessment or counseling^a^	12 (86%)^56–59^ ^,^ ^61–63^ ^,^ ^65–69^
Smoking cessation counseling	3 (21%)^58^ ^,^ ^66^ ^,^ ^68^
Alcohol cessation counseling	3 (21%)^58^ ^,^ ^66^ ^,^ ^68^
Mandatory smoking/alcohol cessation	4 (29%)^57^ ^,^ ^61^ ^,^ ^63^ ^,^ ^67^
Serum albumin measured	2 (14%)^66^ ^,^ ^69^
Post-operative	
Nutritional protocol^b^	10 (71%)^56–58^ ^,^ ^61^ ^,^ ^63–66^ ^,^ ^68^ ^,^ ^69^

a Described explicitly; Not including studies who may have implemented but did not write in protocol.

b Including specific indications when liquids and solids were to be consumed as well as polymeric drinks (n = 8)^57^^,^^58^^,^^61^^,^^63^^,^^64^^,^^66^^,^^68^^,^^69^ and nutritional optimization (n = 1)^65^.

**Table 7.** Nutritional protocols in ERAS for brain tumor surgery (ERABTS)^18^

Author	Year	Country	ERAS patient N	ETOH/smoking cessation	Preoperative nutritional protocol	Preoperative fast (items/hrs pre-op)	Preoperative carbohydrate loading	Postoperative nutritional protocol
Zaed et al.^68^	2023	Switzerland	7 (37%)	SEB, AEB	Not specified; BMI & Weight noted	Not specified	Maltodextrin 100 mL; 2 h	Immediate oral sips (2 h); Personalized postoperative diet
Kaewborisutsakul et al.^61^	2023	Thailand	55 (55%)	MC (4 weeks)	Not specified; BMI noted	Solid food/8	Clear drink 150 mL; 2-4 h	Normal solid diet within 24 h; Water intake within 8 h
Abhinav et al.^56^	2023	India	128 (50%)	Not specified	Support (undescribed)	Not specified	Not specified	*Nutrition*; No description
Jamjoom et al.^60^	2023	UK	21 (47%)	Not specified	Not specified	Not specified	Not specified	Not specified
Khan et al.^62^	2023	USA	63 (34%)	None	Not specified; BMI noted	Not specified	None	Not specified
Pelaez-Sanchez et al.^64^	2023	Spain	76 (100%)	Not specified	Not specified	Not specified	Not specified	Oral liquids & solids within 8 h
Hoffman et al.^59^	2023	USA	202 (51%)	Not specified	Not specified; BMI noted	Not specified	Not specified	Not specified
Sivakumar et al.^65^	2023	USA	164 (100%)	Not specified	Optimization (undescribed)	Not specified	Not specified	Optimization (undescribed)
Wang et al.^67^	2022	China	80 (53%)	MC (not specified)	Baseline assessment (undescribed)	Limited Fasting; No description	Yes, No description	Not specified
Wang et al.^66^	2022	China	76 (50%)	SEB, AEB	Evaluation (undescribed); BMI Noted and Serum Albumin Recorded pre-op	Solid food/6, Clear fluids/2	Oral carbohydrate; 2 h	Oral liquids initiated within 4 h; Full diet at 12-24 h
Elayat et al.^58^	2021	India	35 (50%)	SEB, AEB	Familial involvement (undescribed)	None	Maltodextrin 200 ml; Night before and 100 ml; 2 h	Immediate oral sips within 2 h
Feng et al.^69^	2021	China	50 (50%)	Not specified	Not specified; BMI noted, and Serum Albumin Recorded pre-op	None	Oral 200 mL; 2 h	Oral feeding as early as possible
Chen et al.^57^	2021	China	20 (100%)	MC (2 weeks)	Not specified; BMI noted	Shortened fluid fast; No description	Oral; 2 h	Water intake immediately; Polymeric drink and solid food 4 h
Liu et al.^63^	2020	China	36 (55%)	MC (not specified)	Baseline exam (undescribed)	Not specified	Yes, No description	Early oral liquid/solid intake
			Total N:	1668				

AEB, explained benefits of alcohol cessation; MC, mandatory cessation; SEB, explained benefits of smoking cessation.

a Peri-operative Nutrition Screen with the following criteria: BMI < 18.5 kg/cubic m, unintentional weight loss > 10% in 6 months, serum albumin <3.0 g/dL, poor oral intake for >= 7 days.

Carbohydrate loading was implemented in 9/14 studies (64%). Pre-operative fasting for solid foods was specified in 2 protocols (14%), and likewise, only 2 studies mentioned a shortened fluid fast without detailed specification. As such, beverages and timing varied widely (100-400 mL carbohydrate solutions given 2-4 h preoperatively).

Post-operative Interventions: Ten studies (71%) reported early or immediate oral intake, ranging from sips of water within 2 h to a full diet within 24 h.

Reported Outcomes: The reported clinical outcomes of ERABTS carbohydrate loading are summarized in Table 8. Heterogeneous benefits were observed in studies incorporating carbohydrate loading, including improved ambulation (4/8, 50%), reduced ICU utilization (3/8, 40%), reduced pain (2/8, 25%), and reduced nausea/vomiting (2/8, 25%). Both ERABTS and ERAS studies demonstrated reduced LOS in protocols with or without carbohydrate loading (83% and 37%, respectively). No study reported increased complications directly attributable to nutritional interventions. Despite these apparent benefits, no significant differences were found in overall reductions in hospital LOS (*P* = .08) or cost (*P* = .25) between nutritional protocols that included carbohydrate loading and those that did not.

**Table 8.** Reported oucomes of nutritional and carbohydrates loading protocols in ERABTS^25^

Patient outcome	ERAS nutrition protocols n = 6 (%)	Carb loading protocols n = 8 (%)^a^
Pre-operative		
Reduced thirst	0 (0%)	0 (0%)
Reduced hunger	0 (0%)	0 (0%)
Reduced anxiety	0 (0%)	0 (0%)
Post-operative		
Increased ambulation	1 (17%)^60^	4 (50%)^66–69^
Reduced icu length of stay/utilization	2 (33%)^59^ ^,^ ^62^	3 (40%)^57^ ^,^ ^58^ ^,^ ^68^
Reduced pain	1 (17%)^56^	2 (25%)^66^ ^,^ ^69^
Reduced nausea/vomiting	0 (0%)	2 (25%)^63^ ^,^ ^66^
Reduced opioid usage	0 (0%)	1 (13%)^68^
Increased quality of life	0 (0%)	1 (13%)^63^
Reduced glucose	0 (0%)	0 (0%)
Reduced thirst	0 (0%)	0 (0%)
Reduced time to bowel movement	0 (0%)	0 (0%)
Reduced time to regular diet	0 (0%)	0 (0%)
Reduced insulin resistance	0 (0%)	0 (0%)
Reduced complications	0 (0%)	0 (0%)
Increased wound healing	0 (0%)	0 (0%)
Increased blood lactate	0 (0%)	0 (0%)
Overall		
Reduced length of stay	5 (83%)^56^ ^,^ ^59^ ^,^ ^60^ ^,^ ^62^ ^,^ ^65^	6 (75%)^57^ ^,^ ^61^ ^,^ ^66–69^
Reduced cost of stay	2 (33%)^59^ ^,^ ^65^	3 (38%)^61^ ^,^ ^66^ ^,^ ^68^

a Studies that included carbohydrate loading in their ERABTS Nutritional Protocol.

## Discussion

This study represents the first comparison of nutritional elements within ERAS protocols for general surgery and brain tumor surgery (ERABTS). We found that while nutritional interventions are frequently included, they are inconsistently defined and rarely linked to structured outcome assessment. Carbohydrate loading was widely adopted (86% of ERAS, 64% of ERABTS studies), yet no consistent benefit on length of stay or complications was observed. Importantly, fewer than half of the studies incorporated structured preoperative nutritional screening, and serum albumin was recorded in only 9% of ERAS and 14% of ERABTS studies. These findings underscore the gap between recognition of nutrition as a critical perioperative factor and the absence of standardized approaches in both general and neurosurgical practice. Our findings offer new insight into this emerging field and underscore key knowledge gaps regarding both the assessment of perioperative nutritional status and the role of targeted perioperative nutrition interventions in optimizing patient outcomes. Based on these findings, we propose a novel perioperative nutrition score to identify at-risk patients, enable risk stratification, and facilitate the implementation of tailored nutritional strategies.

International ERAS programs have long emphasized nutrition as a cornerstone of perioperative care. The ERAS Study Group originated from a European nutrition symposium in 2001, and the first formal ERAS Society and implementation programs were launched in Sweden by 2010.^17^ In contrast, the first U.S. ERAS symposium did not occur until 2013, with ERAS USA holding its inaugural formal meeting in 2016.^17^

Neurosurgery has lagged in implementation, with nutritional strategies often vaguely described as “optimization” without defined criteria. Our findings extend prior work identifying nutrition as one of the 8 evidence-based ERABTS elements by systematically quantifying how these interventions have been reported and highlighting the limited outcome data.^18 ^ This study, therefore, provides a foundation for structured protocols that can be validated prospectively.

Carbohydrate loading has clear biologic plausibility.^57^^,^^58^^,^^61^^,^^63^^,^^66–69^ It reduces perioperative insulin resistance, reduces protein catabolism, and preserves skeletal muscle strength, which have been shown to be induced by surgery and preoperative fasting, leading to proteolysis and muscle weakness.^70–73^ Mechanistic studies demonstrate improved handgrip strength, pulmonary function, and glucose homeostasis following preoperative carbohydrate drinks.^70–76^ That said, the risk of an increase in pre-operative blood glucose and post-operative insulin resistance reported in 2 of 19 studies^32^^,^^39^ using carbohydrate loading raises the question of possible increased risk as hyperglycemia has been reported to cause an increase in oxidative stress and inflammation,^77^ as well as reductions in cerebral blood flow and metabolism.^78^

In addition to macronutrient optimization, attention to perioperative electrolyte balance—particularly sodium, potassium, and magnesium—may play an underappreciated role in surgical recovery. Adequate preoperative nutrition helps maintain electrolyte homeostasis, which can influence neuromuscular function, pain modulation, and tissue healing; however, this relationship remains largely unexplored within existing ERAS or ERABTS literature.^9^^,^^79–82^

Yet, our review shows that outcome benefits in ERABTS were inconsistent, mirroring the mixed results in general surgery. Several explanations are possible. First, underpowered studies might be unable to isolate nutritional effects within multimodal ERAS pathways. Second, the heterogeneity in timing, formulation, and dosing of carbohydrate beverages could be a confounding factor. Finally, the inadequate baseline nutritional assessment may blunt the ability to detect subgroup-specific benefits (eg frail or poorly nourished patients).^73^^,^^83^

On the other hand, carbohydrate loading is not universally appropriate. Patients with poorly controlled diabetes, type 1 diabetes, or gastroparesis face heightened risks of perioperative hyperglycemia and aspiration and should therefore be excluded from such protocols.^71^^,^^84^ Moreover, carbohydrate loading does not directly alleviate a high rate of protein catabolism in the post-operative period.

**Figure 2.**
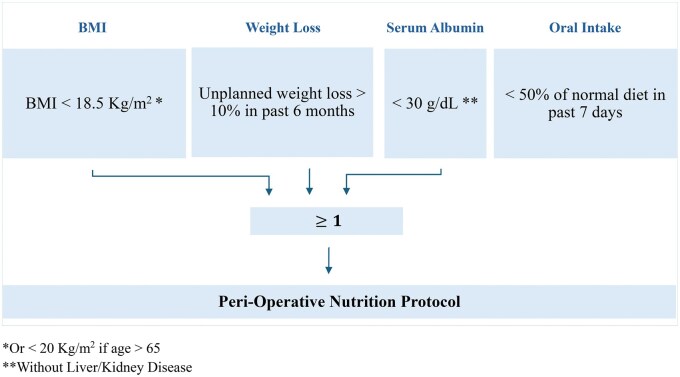
Proposed ERABTS peri-operative nutrition score (PONS). *Or < 20 Kg/m^2^ if age > 65. **Without Liver/Kidney Disease.

The inconsistency in reporting supports the need for standardized assessment. Existing tools such as the malnutrition universal screening tool (MUST)^85^ and the Nutritional Risk Screening 2002 (NRS-2002)^86^ provide general frameworks but do not address neurosurgery-specific challenges. We therefore propose to adopt the perioperative nutrition screen (PONS), which incorporates BMI, weight loss, oral intake, and albumin as a quick screen for at-risk patients^87^ (Figure 2). Such an approach would allow stratification, tailoring of interventions, and prospective validation within ERABTS pathways.

Future studies should prospectively evaluate the PONS as a neurosurgery-specific risk stratification tool within ERABTS pathways. Implementation of PONS could enable early identification of malnourished or metabolically vulnerable patients and guide targeted interventions such as preoperative nutritional supplementation, individualized macronutrient strategies, and intensified postoperative nutritional monitoring. Prospective validation studies should assess the feasibility, interrater reliability, and predictive value of PONS for clinically meaningful outcomes including postoperative complications, length of stay, functional recovery, and health-related quality of life. Integration of PONS into ERAS workflows and electronic health records could facilitate automated screening and real-time clinical decision support, supporting precision perioperative nutrition in neurosurgical populations.

Translating nutritional strategies into neurosurgical practice poses unique challenges. Emergent craniotomies allow little opportunity for preoperative optimization. Cognitive impairment, dysphagia, or altered consciousness may limit adherence to oral protocols. Corticosteroid use, common in brain tumor patients, introduces further metabolic complexity. Recognizing these barriers is essential to realistic protocol design. Early oral intake appears feasible and beneficial, but patient selection remains critical, and protocols must be adaptable to urgent and emergent settings. Additionally, other peri-operative strategies might offer benefit on outcomes. These include increased protein intake that has been shown to attenuate muscle protein breakdown and support nitrogen balance during catabolic stress states.^88^ Furthermore, hyperketonemia—achieved through exogenous ketone supplementation or ketogenic dietary strategies—has been associated with reduced proteolysis, improved metabolic efficiency, and enhanced glycemic control in non-neurosurgical populations.^89^ These approaches may offer theoretical advantages in patients undergoing craniotomy, particularly in the context of corticosteroid exposure and perioperative hyperglycemia.

### Limitations

There are several important limitations to our study that should be acknowledged. The analysis is based on a systematic review of heterogeneous studies, most of which were observational and varied considerably in design, sample size, and reporting standards. The lack of randomized clinical trials (RCTs) limits the ability to draw causal inferences regarding the effectiveness of specific nutritional interventions, including carbohydrate loading. Carbohydrate loading protocols varied substantially, and outcome measures were often embedded within multimodal ERAS bundles, making it difficult to isolate nutritional effects. Further, potential confounding from institutional practices, patient selection, and regional differences in perioperative care could not be fully controlled. Our exclusion of diabetic populations, while necessary to reduce confounding, limits generalizability. Almost all the ERAS protocols evaluated do not include cancer patients. Nutritional needs in brain tumor and brain cancer patients may vary based on their disease state and recent adjuvant therapies. No formal risk-of-bias assessment was conducted, consistent with the descriptive scope of this review, though this remains a limitation.

Additionally, tumor characteristics (tumor size/volume, tumor location, etc.) and treatment variables (steroid use, chemotherapy use, etc.) can significantly impact patient outcomes, and undoubtedly, patient nutritional needs. These variables are not sufficiently granular in the currently published literature to allow further stratification. Finally, the proposed PONS score is derived from pooled evidence and expert interpretation rather than prospective validation. This review is subject to inherent limitations of the available data, including publication bias, English-language restriction, and the possibility that relevant studies were missed despite a comprehensive search strategy. Together, these factors highlight the need for prospective, and standardized investigations to confirm the role of perioperative nutritional assessment and to clarify the independent contributions of specific nutritional interventions within ERABTS pathways.

## Conclusions

This systematic review demonstrates that while nutritional interventions are frequently included in ERAS and ERABTS protocols, they are inconsistently defined and rarely linked to structured outcomes. Carbohydrate loading was widely used but showed no consistent independent benefit on length of stay or complications, underscoring the need for more rigorous evaluation. To address these gaps, we propose a Perioperative Nutrition Score (PONS) to identify at-risk patients, enable risk stratification, and guide tailored interventions in brain tumor surgery. Prospective validation of standardized nutritional protocols, including PONS, will be essential to optimize recovery and outcomes in neurosurgical oncology.

## Data Availability

The scoping review data that support the study findings are available from the corresponding author upon reasonable request.
